# Endolysosomal N-glycan processing is critical to attain the most active form of the enzyme acid alpha-glucosidase

**DOI:** 10.1016/j.jbc.2021.100769

**Published:** 2021-05-08

**Authors:** Nithya Selvan, Nickita Mehta, Suresh Venkateswaran, Nastry Brignol, Matthew Graziano, M. Osman Sheikh, Yuliya McAnany, Finn Hung, Matthew Madrid, Renee Krampetz, Nicholas Siano, Anuj Mehta, Jon Brudvig, Russell Gotschall, Jill M. Weimer, Hung V. Do

**Affiliations:** 1 Discovery Science Division, Amicus Therapeutics, Inc., Philadelphia, Pennsylvania, USA; 2 Pediatrics & Rare Diseases Group, Sanford Research, Sioux Falls, South Dakota, USA

**Keywords:** glycogen storage disease, lysosomal glycoprotein, lysosomal storage disease, lysosome, enzyme processing, mannose 6-phosphate, AOAA, aminooxyacetic acid, BSA, bovine serum albumin, CHO, Chinese hamster ovary, CI-MPR, cation-independent M6P receptor, EIC, extracted ion chromatogram, Endo H, Endoglycosidase H, ERT, enzyme replacement therapy, FBS, fetal bovine serum, GAA, acid alpha-glucosidase, HCD MS2, higher-energy collisional dissociation MS2, HPAEC-PAD, high-pH anion-exchange chromatography with pulsed amperometric detection, kif, kifunensine, LSD, least significant difference, M6P, mannose 6-phosphate, MW, molecular weight, MWCO, molecular weight cutoff, PNGase F, peptide:N-glycanase F, rhGAA, recombinant human GAA, TBS, tris buffered saline, WGA, wheat germ agglutinin

## Abstract

Acid alpha-glucosidase (GAA) is a lysosomal glycogen-catabolizing enzyme, the deficiency of which leads to Pompe disease. Pompe disease can be treated with systemic recombinant human GAA (rhGAA) enzyme replacement therapy (ERT), but the current standard of care exhibits poor uptake in skeletal muscles, limiting its clinical efficacy. Furthermore, it is unclear how the specific cellular processing steps of GAA after delivery to lysosomes impact its efficacy. GAA undergoes both proteolytic cleavage and glycan trimming within the endolysosomal pathway, yielding an enzyme that is more efficient in hydrolyzing its natural substrate, glycogen. Here, we developed a tool kit of modified rhGAAs that allowed us to dissect the individual contributions of glycan trimming and proteolysis on maturation-associated increases in glycogen hydrolysis using *in vitro* and *in cellulo* enzyme processing, glycopeptide analysis by MS, and high-pH anion-exchange chromatography with pulsed amperometric detection for enzyme kinetics. Chemical modifications of terminal sialic acids on N-glycans blocked sialidase activity *in vitro* and *in cellulo*, thereby preventing downstream glycan trimming without affecting proteolysis. This sialidase-resistant rhGAA displayed only partial activation after endolysosomal processing, as evidenced by reduced catalytic efficiency. We also generated enzymatically deglycosylated rhGAA that was shown to be partially activated despite not undergoing proteolytic processing. Taken together, these data suggest that an optimal rhGAA ERT would require both N-glycan and proteolytic processing to attain the most efficient enzyme for glycogen hydrolysis and treatment of Pompe disease. Future studies should examine the amenability of next-generation ERTs to both types of cellular processing.

Acid alpha-glucosidase (GAA) is a soluble acid hydrolase that catabolizes glycogen in lysosomes (1). Approximately three-fourths of the body’s total glycogen is stored in muscles (2), but unlike cytoplasmic glycogen that has a well-defined biological function as an energy source, the role of lysosomal glycogen has not yet been elucidated. Nonetheless, GAA plays an essential role in glycogen metabolism to help maintain cellular homeostasis in many cells, particularly in cardiomyocytes and skeletal muscle cells. Biallelic pathogenic mutations in *GAA* result in Pompe disease (glycogen storage disease type II), a phenotypically variable disorder characterized by lysosomal glycogen accumulation, progressive muscle weakness, and respiratory insufficiency. The most severe cases have infantile onset and typically result in death by 1 year of age because of cardiac failure (3, 4). Late-onset cases are characterized by low residual GAA activity and milder disease, but these cases still result in significant disability, with progressive muscle weakness often leading to loss of mobility and dependence on a ventilator (5, 6). Late-onset patients also face the prospect of early mortality because of respiratory failure.

Existing treatments for Pompe disease intend to restore lysosomal GAA activity through enzyme replacement therapy (ERT). As with many other enzymes targeted to the lysosome, endogenous GAA is post-translationally modified with mannose 6-phosphate (M6P), which facilitates high-affinity binding to cation-dependent M6P receptors and cation-independent M6P receptor (CI-MPR), and enables transport to lysosomes from the *trans*-Golgi network (7, 8, 9). The CI-MPR also cycles to the plasma membrane, where it facilitates endocytosis and lysosomal targeting of M6P-bearing exogenous lysosomal enzymes, including recombinant human GAA (rhGAA) (7, 10, 11, 12). It is this natural endocytic CI-MPR pathway that is exploited for most lysosomal ERT.

ERT is an unnatural process whereby recombinant lysosomal enzymes are infused into the circulation and requires the exogenous enzyme to be internalized into affected target cells to supplement the deficient activity. Several factors make lysosomal ERT targeting particularly difficult for rhGAA. The most problematic issue is that only a small fraction of intravenously delivered rhGAA reaches the interstitial space such that very low resultant ERT concentrations (estimated to be in tens of nanomolar range) are available for uptake in targeted muscle cells (estimated to be <1% of total enzyme dose) (13). It is therefore critical for rhGAA to be efficiently internalized *via* the CI-MPR pathway under these low enzyme conditions. However, the current U.S. Food and Drug Administration approved rhGAA ERT has a low abundance of M6P, particularly bisphosphorylated high-mannose N-glycans (bearing two M6Ps on same N-glycan), which severely limits cellular uptake of ERT at low interstitial enzyme concentrations. Bisphosphorylated N-glycans are known to have the highest binding affinity of all known carbohydrate structures for the CI-MPR and enable binding at low nanomolar concentrations (12, 14). Approximately only 1% of N-glycans on the standard-of-care rhGAA ERT bear *bis*-M6P (13, 15). This ERT contains predominantly monophosphorylated glycans that are known to have approximately 3000-fold lower affinity for CI-MPR than bisphosphorylated N-glycans (14, 15). Because monophosphorylated N-glycans cannot bind the CI-MPR at low nanomolar enzyme concentrations, this leads to inefficient cellular uptake and lysosomal delivery (14). Cells from patients with Pompe disease also have defects in CI-MPR trafficking and surface expression (16), compounding these difficulties in target muscle cells, where pathology is often widespread at the time of treatment.

In the face of these limitations, it is critical for the small amount of rhGAA that is internalized in cells to exhibit optimal glycogen hydrolytic activity once in lysosomes. Endolysosomal processing appears to be essential for attaining peak activity (10, 17). After endolysosomal delivery, the single-chain, 110-kDa GAA precursor undergoes sequential proteolytic processing to attain the mature 76/70 kDa forms (18). Glycan trimming also takes place within the endolysosomal system, whereby terminal monosaccharides are sequentially removed resulting in approximately five monosaccharides per glycan in the mature form as opposed to 9 to 11 monosaccharides per glycan in the precursor form (18). The mature GAA form has been reported to exhibit a 10-fold higher affinity for glycogen (10, 17), suggesting that processing events may remove amino acids or carbohydrate moieties that sterically hinder the relatively narrow active site or the secondary carbohydrate-binding domain (19). However, the concomitant nature of these proteolytic and glycan trimming events has made it difficult to understand their individual contributions toward increased enzyme activity and affinity for glycogen.

Although structural studies of rhGAA define a role for proteolytic processing in GAA maturation (19), there are reasons to believe that glycan trimming may also be important. GAA contains seven potential N-glycosylation sites, six of which are always glycosylated, whereas a single site near the C terminus is glycosylated approximately 50% of the time (18). One of these, site 5 (N652), is proximal to the active site of the enzyme (19), and bulky untrimmed glycan structures here could potentially hinder substrate interactions.

Here, we developed a tool kit of modified rhGAAs that allowed us to individually dissect the relative contributions of glycan trimming and proteolysis on glycogen hydrolytic activity. First, we chemically modified terminal sialic acids on rhGAA N-glycans to block glycan trimming. Because N-glycan trimming proceeds in a sequential manner through the action of specific exoglycosidases within lysosomes, we hypothesized that blocking cleavage of the terminal sialic acid residue would also block all subsequent glycan trimming of sialylated complex-type glycans, without affecting proteolysis. This would enable us to assess the impact of proteolytic processing for improving GAA activity. We also generated an rhGAA with N-glycan structures that could be enzymatically removed *in vitro* without affecting its native protein structure or enzyme activity. This allowed us to assess the effects of glycan trimming on GAA activation without concomitant proteolytic cleavage. Together, these tools allowed us to discern the relative impacts of proteolysis and glycan trimming on the glycogen hydrolytic activity of GAA.

## Results

### Synthesis of modified rhGAAs

To investigate the relative contributions of glycan trimming and proteolytic processing on GAA maturation, we developed a tool kit of modified rhGAAs that would allow us to assess these processes independently. First, we modified sialylated complex-type N-glycans, which are the most abundant N-glycan structures on rhGAA (11), in such a manner that would render them nonhydrolyzable. The glycerol moiety extending from C7 of terminal sialic acid is known to be involved in substrate recognition by exosialidases through enzyme side-chain interactions with O7 and O9 oxygens on the sugar (20). We hypothesized that removing this moiety by oxidation or replacing it with aminooxyacetic acid (AOAA) would disrupt substrate binding or positioning of the glycosidic bond for hydrolysis, and thereby block exosialidase activity. Given the sequential nature of glycan processing by various resident lysosomal exoglycosidases, we reasoned that blocking sialidase activity would also block the activities of downstream exoglycosidases by impeding access to their substrates, thereby interrupting all processing of sialylated glycans without impacting the processing of nonsialylated glycans or proteolytic processing.

**Figure 1:**
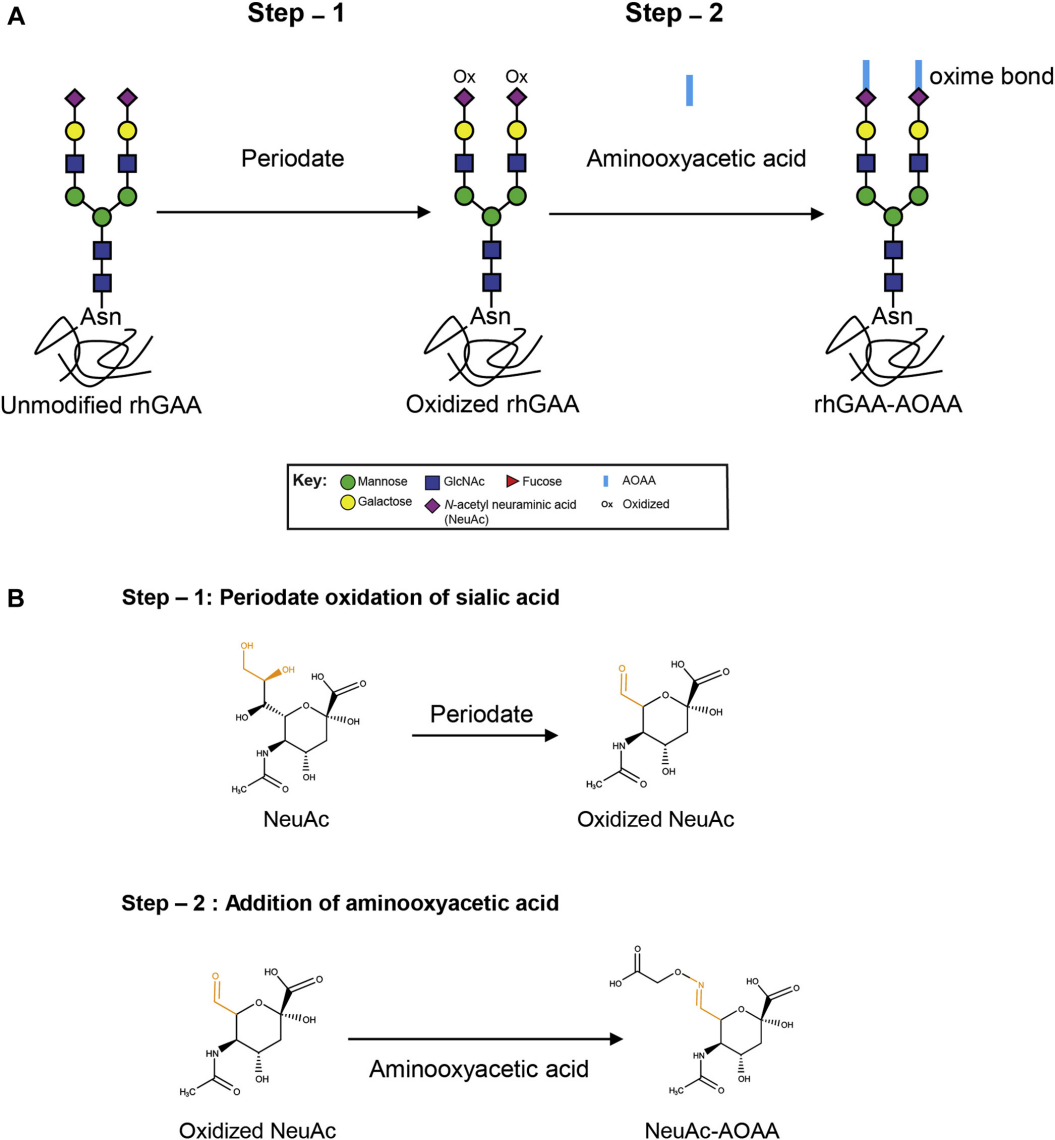
**Synthesis of rhGAAs containing modified terminal sialic acids.***A*, a schematic of chemical modification of terminal sialic acids on rhGAA complex N-glycan structures. rhGAA is oxidized with sodium metaperiodate in the first step of synthesis. Oxidized rhGAA is then reacted with AOAA to obtain rhGAA–AOAA. Glycan annotations are represented by symbols according to Symbol Nomenclature for Glycans (SNFG) conventions. *B*, chemical structures of modified sialic acid. Step 1 of the synthesis showing periodate oxidation of sialic acid at C7 to form an aldehyde. Oxidized sialic acid is further modified in the second step with AOAA to form a conjugate that contains an oxime bond. AOAA, aminooxyacetic acid; GAA, acid alpha-glucosidase; rhGAA, recombinant human GAA.

Oxidation of sialic acid with sodium periodate followed by aniline-catalyzed oximation to introduce aminooxy functional groups to C7 was utilized for this purpose because this approach is well established for applications such as solid support immobilization and tag conjugation (21, 22, 23). First, we treated rhGAA with 2 mM (24, 25) or 10 mM sodium metaperiodate to oxidize terminal sialic acid/*N*-acetylneuraminic acid (Fig. 1, *A* and *B*). High-pH anion-exchange chromatography with pulsed amperometric detection (HPAEC-PAD) revealed that 2 mM sodium metaperiodate treatment only partially oxidized terminal sialic acids (∼30%), whereas 10 mM sodium metaperiodate oxidized >90% of terminal sialic acids (data not shown). We therefore utilized rhGAA samples with >90% oxidized sialic acids for subsequent reactions with AOAA to obtain rhGAA–AOAA (Fig. 1, *A* and *B*) with oxime-bonded AOAA at C7.

**Figure 2:**
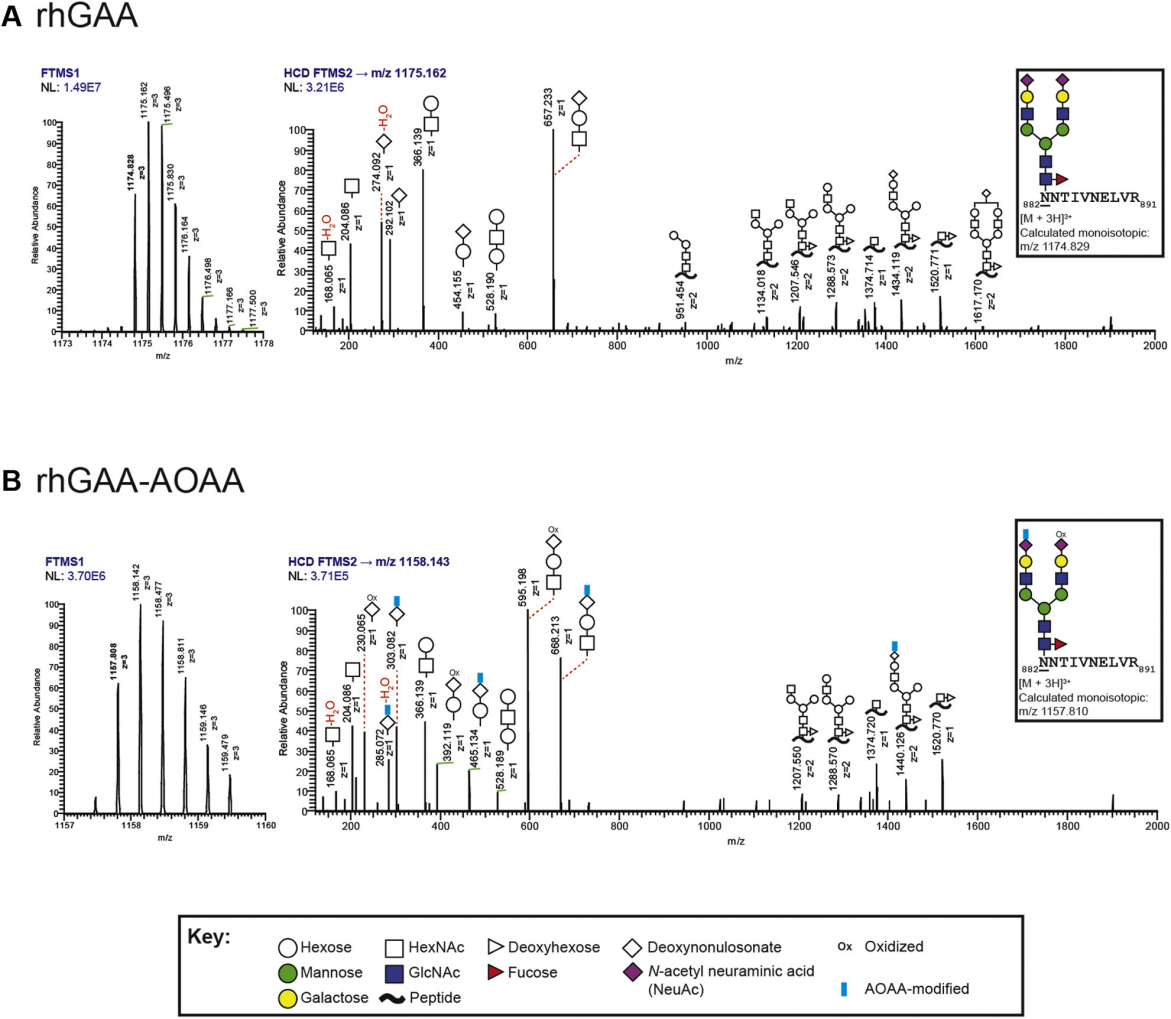
**Glycopeptide analysis of rhGAA and rhGAA–AOAA by MS.** Unmodified and modified rhGAAs were subjected to trypsinization followed by glycopeptide analysis by LC-MS. Representative precursor MS1 and HCD MS2 spectra of the rhGAA tryptic glycopeptide, _882_NNTIVNELVR_891_, bearing the highly abundant glycan structure FA2G2S2 at Asn882 (*underlined*), are shown for unmodified (*A*) and AOAA-modified (*B*) rhGAA. Glycan annotations are represented by symbols according to SNFG conventions. Glycan oxonium ions are annotated in the region 150 to 800 *m/z*. Fragment ions consistent with various glycosidic bond cleavages of the glycan attached to the peptide are also annotated. Calculated monoisotopic *m/z* values are displayed, and the observed monoisotopic *m/z* values are *bolded* in the MS1 spectra. [FTMS (Orbitrap); HCD; NL; *m/z*]. *A*, the MS1 and HCD MS2 spectra are consistent with the glycan structure FA2G2S2, with oxonium ions 292 and 274 *m/z* indicative of a glycan species containing sialic acid. *B*, HCD MS2 spectra for FA2G2S2 in rhGAA–AOAA contains fragments consistent with only one of the two oxidized sialic acids further derivatized with AOAA (FA2G2S2, NeuAcAOAA+NeuAcOxi). Unique oxonium ions were detected that are consistent with the oxidation of sialic acids (230 and 392 *m/z*) or the modification of sialic acids with AOAA (285, 303, 465, and 668 *m/z*). AOAA, aminooxyacetic acid; FTMS, Fourier transform mass spectrometry; GAA, acid alpha-glucosidase; HCD, higher-energy collision dissociation; HCD MS2, higher-energy collisional dissociation MS2; NL, normalization level, signal intensity of base peak; rhGAA, recombinant human GAA; SNFG, Symbol Nomenclature for Glycans.

We examined the efficiency of AOAA conjugation *via* glycopeptide analysis by LC coupled to high-resolution tandem MS. An unbiased analysis of the glycan structures at each of the seven N-glycan sites on unmodified rhGAA revealed greater glycan microheterogeneity than previously reported (11, 26), although the most abundant glycan structures observed were consistent with prior reports (11) (Fig. S1). Examination of rhGAA–AOAA revealed that ∼4 mol of AOAA was added per mol of rhGAA (Table S1, Fig. S2), in agreement with published reaction efficiencies (25). As a representative example, we analyzed precursor (MS1) and higher-energy collisional dissociation MS2 (HCD MS2) spectra of the rhGAA tryptic glycopeptide, _882_NNTIVNELVR_891_, bearing the highly abundant glycan structure FA2G2S2 at Asn882 [glycan structure nomenclature according to the Oxford convention, where F indicates a core fucose, Ax where x is the number of antenna (GlcNAc) on the trimannosyl core of the complex N-glycan structure, Gx, where x is the number of linked galactose, and Sx, where x is the number of sialic acids linked to galactose] (Fig. 2, *A* and *B*). The LC-MS spectra confirmed the presence of two terminal sialic acids on this glycopeptide derived from unmodified rhGAA (Fig. 2*A*). For the corresponding glycopeptide from rhGAA–AOAA, spectra revealed that both terminal sialic acids were oxidized, but only one of the two oxidized sialic acids were further modified with AOAA (Fig. 2*B*). Nonetheless, these LC-MS data indicate that there is sufficient modification of terminal sialic acids for assessing potential downstream impacts on glycan processing.

**Figure 3:**
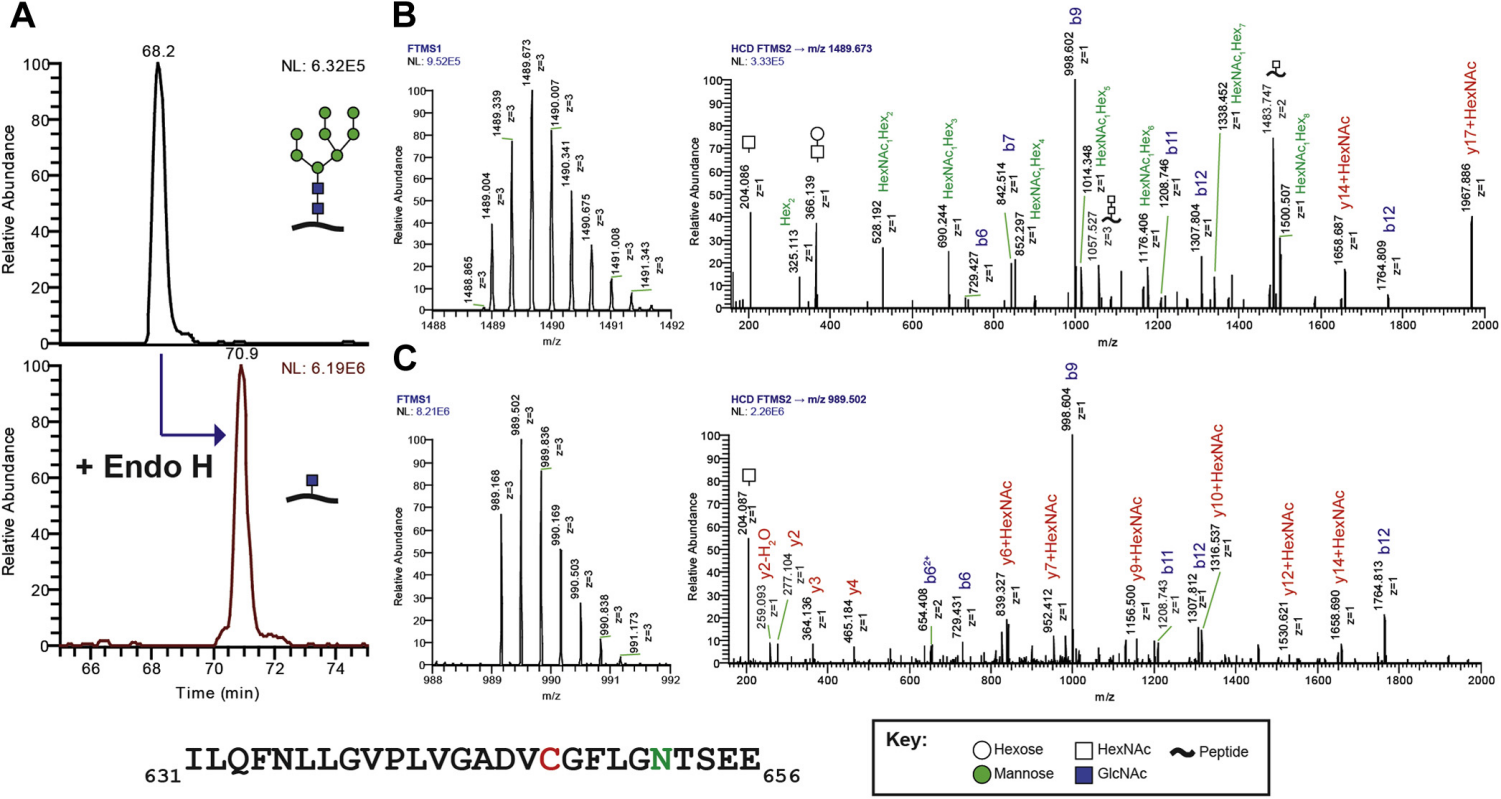
**Oligomannose N-glycans at site 5 (N652) of rhGAA–kif are removed with Endo H treatment.** Recombinant human GAA expressed in cells treated with the mannosidase inhibitor kifunensine were treated with Endo H *in vitro* and subjected to proteolytic digestion using GluC followed by glycopeptide analysis by LC-MS. Glycan annotations are represented by symbols according to SNFG conventions. For the peptide sequence shown at the *bottom*, *green* indicates the site of N-glycosylation at Asn 652 and *red* indicates carbamidomethylated Cys residues. Peptide backbone fragmentation is annotated on the spectra using the standard b/y ion convention. *A*, extracted ion chromatograms (EICs) of the ions 1489.67 and 989.50 *m/z* corresponding to the observed triply charged glycopeptide species for site 5 bearing a Man_8_GlcNAc_2_ or single GlcNAc, respectively. The glycopeptide, _631_ILQFNLLGVPLVGADVCGFLGNTSEE_656_, is modified at Asn 652 (*underlined*) by the N-glycans mentioned above. Untreated rhGAA–kif is shown on *top* and Endo H–treated is shown on the *bottom*. *B*, MS1 and HCD MS2 spectra for untreated rhGAA–kif. *C*, MS1 and HCD MS2 spectra for Endo H–treated rhGAA–kif. GAA, acid alpha-glucosidase; HCD MS2, higher-energy collisional dissociation MS2; kif, kifunensine; rhGAA, recombinant human GAA; SNFG, Symbol Nomenclature for Glycans.

We also sought to generate a form of rhGAA that could undergo glycan trimming in the absence of any proteolytic processing. To achieve this, we first expressed rhGAA in the presence of the alpha-mannosidase inhibitor kifunensine (kif) and purified the secreted 110-kDa precursor GAA referred to here as rhGAA–kif, which primarily contained high mannose-type N-glycans (27). As expected, LC-MS analysis confirmed that rhGAA–kif contained high-mannose-type N-glycans on sites 1 to 5 of seven possible N-glycan sites (Fig. S3). Representative HCD MS2 spectra for the glycopeptide _631_ILQFNLLGVPLVGADVCGFLGNTSEE_656_ containing Asn 652 (site 5), which is proximal to the active site of GAA, demonstrate the presence of the glycan structure M8 (where Mx represents the number of mannose residues according to the Oxford convention) (Fig. 3, *A* and *B*). Sites 6 and 7 still were found to contain complex N-glycans in addition to high-mannose-type N-glycans (Fig. S3).

We then subjected rhGAA–kif to *in vitro* deglycosylation by treatment with Endoglycosidase H (Endo H), which cleaves between the two GlcNAc residues of the chitobiose core of high-mannose-type N-glycans, leaving a single GlcNAc attached to Asn (28). Endo H treatment resulted in a single N-linked GlcNAc being the most abundant structure at all seven sites, although complex glycans were also observed at sites 6 and 7 at lower abundances (Fig. S4). A notable and expected shift in retention time was also observed between the untreated and Endo H–treated glycopeptides for site 5 (extracted ion chromatogram (EIC) with a shift in retention time compared with the corresponding untreated glycopeptide from rhGAA–kif and precursor MS1 and HCD MS2 spectra for the Endo H–treated glycopeptide containing Asn 652 bearing a single GlcNAc residue are shown in Figure 3, *A*–*C*). MS analysis confirmed that no proteolytic cleavage occurred after purification of the rhGAA–kif precursor, with peptide coverage obtained for both termini (data not shown). The major advantage of this approach was that we were able to remove N-glycans without denaturing the protein or altering enzyme activity for studying the direct impact of glycan trimming independent of protein processing for GAA activation.

### Oxidized rhGAA and rhGAA–AOAA are resistant to sialidase activity, *in vitro*

**Figure 4:**
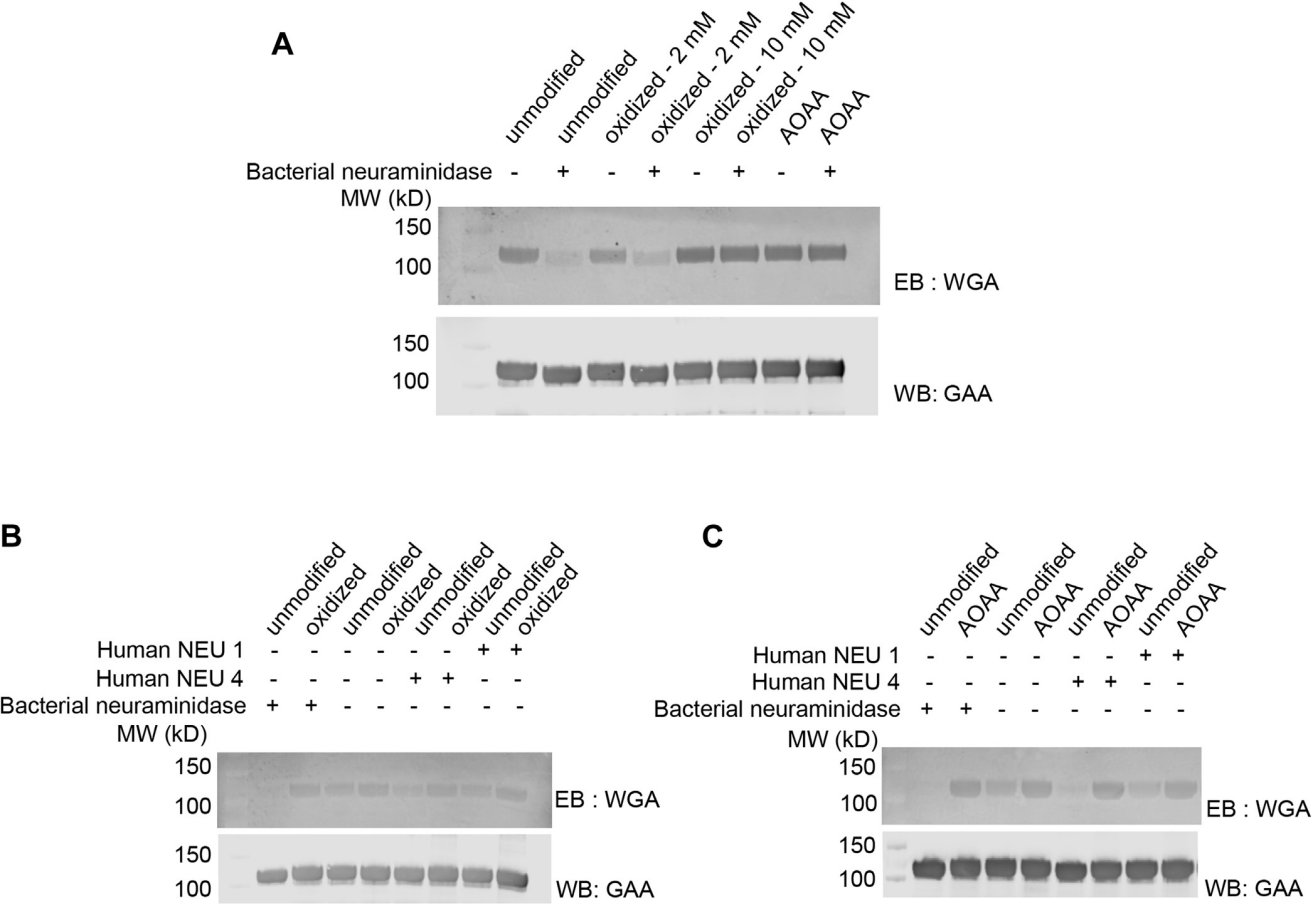
**C7-modified sialic acid is resistant to sialidases.** Unmodified rhGAA, oxidized rhGAA, and rhGAA–AOAA were treated with bacterial neuraminidase or the human lysosomal sialidases NEU4 and NEU1, and blots were probed with the lectin wheat germ agglutinin (WGA), which binds to terminal sialic acids. Blots are probed with an anti-GAA antibody as a loading control. *A*, a reduced WGA signal is observed for rhGAA and 30% oxidized rhGAA after treatment with bacterial neuraminidase, but similar effects are not observed for 90% oxidized rhGAA or rhGAA–AOAA. *B*, treatment of unmodified rhGAA with bacterial sialidase or human NEU1 or NEU4 led to a reduction in the WGA signal; however, treatment of oxidized rhGAA with bacterial sialidase or human NEU1 or NEU4 did not lead to a reduction WGA signal. *C*, treatment of unmodified rhGAA but not rhGAA–AOAA with bacterial sialidase or human NEU1 or NEU4 led to a reduction WGA signal. AOAA, aminooxyacetic acid; GAA, acid alpha-glucosidase; rhGAA, recombinant human GAA.

Human sialidases exhibit a high degree of substrate specificity (20, 29), suggesting that unnatural substrate conjugates on sialic acids could interfere with substrate recognition and hydrolysis. To investigate this, we examined whether chemical modifications to C7 of terminal sialic acids impact sialidase activity. Sialidase activity can be monitored by Eastern blotting with wheat germ agglutinin (WGA), which recognizes terminal sialic acids. Unmodified precursor rhGAA, 30% oxidized rhGAA, 90% oxidized rhGAA, and rhGAA–AOAA all exhibited a robust WGA signal as expected given that WGA binds to sialic acids through the C2 through C4 positions of the pyranose ring (30, 31) (Fig. 4*A*), which were not modified under our reaction conditions. Treatment of unmodified rhGAA with bacterial neuraminidase resulted in a drastic reduction in the WGA signal, demonstrating that the vast majority of unmodified sialic acids are removed from rhGAA under these *in vitro* experimental conditions. When modified rhGAA glycoforms were subjected to the same neuraminidase treatment and WGA blotting, the 30% oxidized GAA sample exhibited some reduction in the WGA signal, suggesting that unmodified sialic acids were removed by the bacterial neuraminidase but the oxidized sialic acids were retained and detected by WGA. This hypothesis is supported by the 90% oxidized rhGAA and rhGAA–AOAA samples that did not exhibit any apparent reduction, suggesting that these C7 chemical modifications efficiently blocked bacterial neuraminidase activity.

To test whether modified sialic acids similarly block human lysosomal sialidase activities, we repeated the assay using purified recombinant human neuraminidases NEU1 and NEU4 (32, 33). The human enzymes exhibited similar activities against the synthetic fluorogenic substrate 2′-(4-methylumbelliferyl)-α-D-N-acetylneuraminic acid *in vitro* (data not shown), but differed in their ability to hydrolyze sialic acids on rhGAA, with NEU4 exhibiting efficient hydrolysis and NEU1 exhibiting lower (albeit still detectable) levels of activity. We speculate that may be due in part to the necessity for NEU1 to complex with cathepsin A and beta-galactosidase for full activity (34, 35) (Fig. 4, *B* and *C*). Importantly, NEU4 and NEU1 activity was also shown to be blocked for the 90% oxidized rhGAA (Fig. 4*B*) and rhGAA–AOAA samples (Fig. 4*C*), with no perceptible decrease in the WGA signal, indicating that either type of C7 chemical modification disrupts human neuraminidase activity.

**Figure 5:**
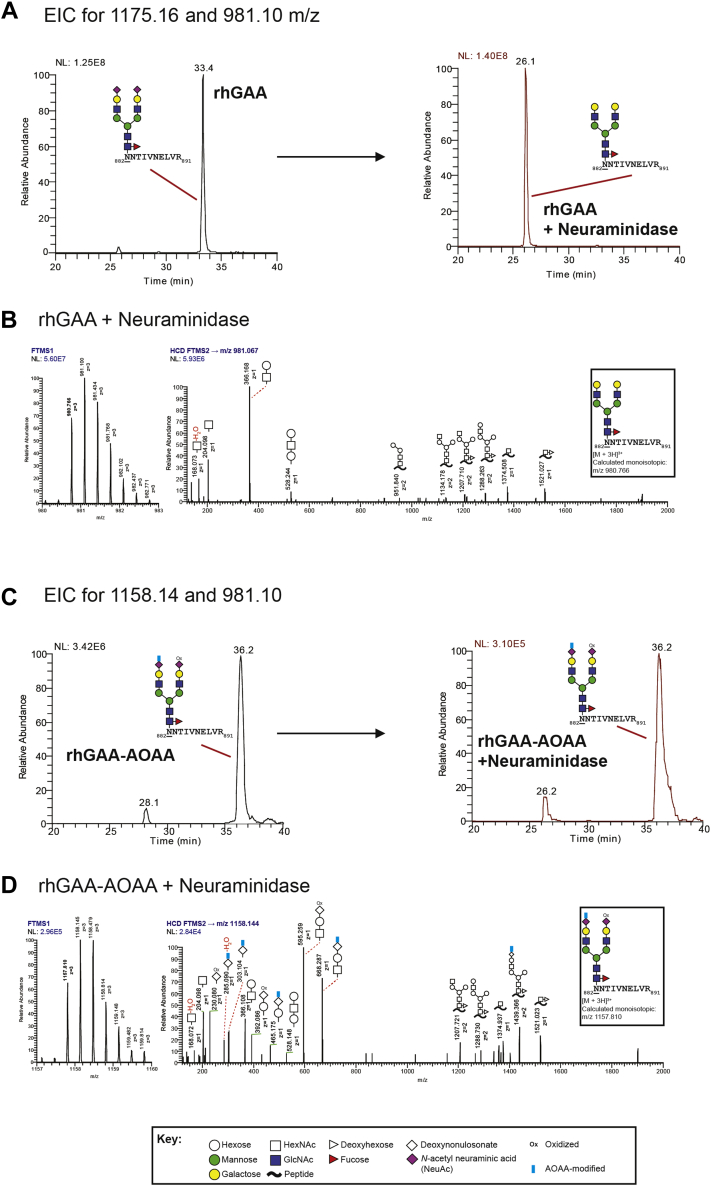
**C7-modified sialic acid is resistant to neuraminidase activity.** Unmodified and modified rhGAAs were treated with bacterial neuraminidase *in vitro* and subjected to trypsinization followed by glycopeptide analysis by LC-MS. Representative precursor MS1 and HCD MS2 spectra of the highly abundant rhGAA tryptic glycopeptide, _882_NNTIVNELVR_891_, bearing the glycan structure FA2G2S2 at Asn882 (*underlined*), are shown. Glycan annotations are represented by symbols according to SNFG conventions. Glycan oxonium ions are annotated in the region 150 to 800 *m/z*. Fragment ions consistent with various glycosidic bond cleavages of the glycan attached to the peptide are also annotated. Calculated monoisotopic *m/z* values are displayed, and the observed monoisotopic *m/z* values are *bolded* in the MS1 spectra. [FTMS (Orbitrap); HCD; NL; *m/z*; EIC]. *A*, EICs of the ions 1175.16 and 981.10 *m/z* corresponding to the observed triply charged glycopeptide species mentioned above containing terminal sialic acid (FA2G2S2) or terminal galactose (FA2G2), respectively. Unmodified rhGAA is shown on the *left*, whereas neuraminidase-treated rhGAA is shown on the *right*. Neuraminidase treatment results in a dramatic increase in the detection of the FA2G2 species at 26 min in comparison with FA2G2S2, which is detected at 33 min. *B*, representative MS1 and HCD MS2 spectra of the neuraminidase-treated rhGAA glycopeptide. _882_NNTIVNELVR_891_, is consistent with the glycan structure FA2G2. *C*, EICs of the ions 1158.14 and 981.10 *m/z* corresponding to the observed triply charged glycopeptide species mentioned above containing terminal sialic acids (designated FA2G2S2, NeuAcAOAA+NeuAcOxi; one oxidized and one modified with AOAA) or terminal galactose (FA2G2), respectively. rhGAA–AOAA is shown on the *left*, whereas neuraminidase-treated rhGAA–AOAA is shown on the *right*. Neuraminidase treatment does not result in an increase in the detection of the FA2G2 species as demonstrated in panel *A* with unmodified rhGAA nor did it result in reduction of FA2G2S2 (NeuAcAOAA+NeuAcOxi) species at 36 min. *D*, representative MS1 and HCD MS2 spectra of the neuraminidase-treated rhGAA–AOAA glycopeptide of interest. Unique oxonium ions were detected that are consistent with the oxidation of sialic acids (230 and 392 *m/z*) or the modification of sialic acids with AOAA (285, 303, 465, and 668 *m/z*). AOAA, aminooxyacetic acid; EIC, extracted ion chromatogram; FTMS, Fourier transform mass spectrometry; GAA, acid alpha-glucosidase; HCD, higher-energy collision dissociation; NL, normalization level, signal intensity of base peak; rhGAA, recombinant human GAA; SNFG, Symbol Nomenclature for Glycans.

For an orthogonal analysis of exoneuraminidase activity, untreated and bacterial neuraminidase-treated rhGAA samples were also analyzed by LC-MS, again focusing on the most abundant glycan on the tryptic peptide _882_NNTIVNELVR_891_ (Figs. S5–S8). EICs demonstrated that FA2G2S2 (peak detected at 33 min) is the predominant glycan species present in the glycopeptide derived from unmodified precursor rhGAA before neuraminidase treatment (Fig. 5*A*). EICs revealed a significant peak shift after bacterial neuraminidase treatment of unmodified rhGAA with a predominance of the FA2G2 glycan structure (detected at 26 min, Fig. 5, *A* and *B*), confirming efficient removal of both terminal sialic acids. In stark contrast, no shift was observed for rhGAA–AOAA after neuraminidase treatment, confirming that C7-modified terminal sialic acid is refractory to neuraminidase activity, *in vitro* (Fig. 5, *C* and *D*).

### Blocking sialidase activity abolishes rhGAA glycan trimming in cellulo

Owing to the sequential, “outside-in” nature of lysosomal glycan trimming by exoglycosidases, we asked whether abolishing sialidase activity on rhGAA would also prevent downstream glycan trimming events *in cellulo*. Similarly, we asked whether blocking glycan trimming would impact GAA protein processing. To investigate these questions, we performed cellular uptake studies using fibroblasts derived from patients with Pompe disease to enable natural processing of GAA within the endolysosomal system. We utilized fibroblasts derived from patients with Pompe disease because they have dramatically reduced endogenous GAA levels to enable characterization of exogenous rhGAA (36). Cells were incubated with 500 nM unmodified rhGAA, oxidized rhGAA, or rhGAA-AOAA for 16 h. After cellular uptake, cells were washed and incubated further in normal growth media. Importantly, because the chemical modifications of sialic acids occurred only on complex-type N-glycans, these modifications do not affect rhGAA cellular uptake that is mediated by M6P-bearing high-mannose N-glycans. When treated in this manner, fibroblasts internalize exogenous M6P-bearing rhGAA *via* the CI-MPR and shuttle it through the endocytic pathway to lysosomes, where proteolytic and glycan trimming events take place. Cells were then harvested at different time points and analyzed by Western blotting using a monoclonal anti-human GAA primary antibody (raised against a synthetic peptide immunogen from the N terminus of precursor human GAA comprising amino acids 150–250). This antibody detects the N-terminal fragment of GAA isoforms including the precursor (110 kDa) and intermediate processed (95 kDa and 76 kDa) forms of GAA. The 70-kDa mature GAA isoform is not detected by this antibody; rather, the antibody detects the ∼10-kDa N-terminal fragment that is proteolytically removed from the larger catalytic fragment. This N-terminal ∼10-kDa fragment is known to be associated in a very tight complex with the larger catalytic fragment and the C-terminal fragment in native/nondenaturing conditions (18).

**Figure 6:**
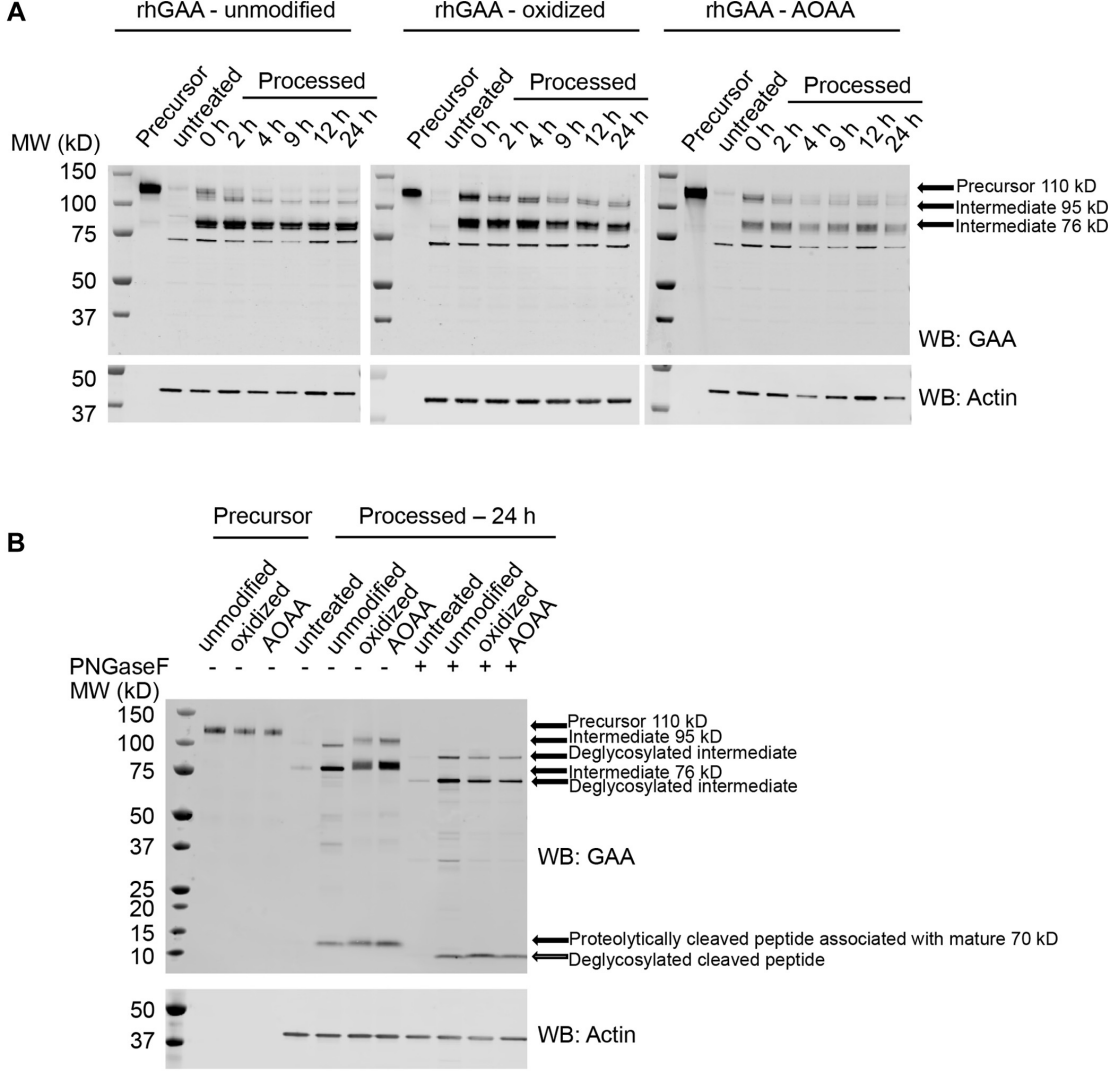
**rhGAA and modified rhGAAs are differentially processed after internalization in fibroblasts of patients with Pompe disease**. Fibroblasts of patients with Pompe disease were treated with 500 nM unmodified rhGAA, oxidized rhGAA, or rhGAA–AOAA for 16 h at 37 °C. Uptake media were replaced by growth media after 16 h, and cells were harvested at the indicated time points, lysed, and analyzed by Western blotting. *A*, Western blot analysis of unmodified rhGAA, oxidized rhGAA, and rhGAA–AOAA processed *in cellulo* over a 24-h time course compared with precursor enzyme (lane 1 of each blot) probed with a primary antibody against GAA (*top*) or actin (*bottom*). Precursor and processed bands are highlighted by *arrows*, and time points of harvest are indicated above each lane. *B*, to differentiate between proteolytic and glycan processing, a PNGase F digest was performed on lysates harvested at 24 h, and Western blots were probed with a primary antibody against GAA (*top*) or actin (*bottom*). PNGase F digested (*right*) and undigested lysates (*middle*) were compared with unprocessed enzymes (*left*). AOAA, aminooxyacetic acid; GAA, acid alpha-glucosidase; PNGase F, peptide:N-glycanase F; rhGAA, recombinant human GAA.

The cellular uptake studies showed that all rhGAA test samples were internalized and underwent proteolysis, with the ∼76-kDa cleavage product apparent in cell lysates collected immediately after the 16-h uptake period (Fig. 6*A*). Unmodified rhGAA also underwent glycan trimming over the next 9 h as evidenced by a gradual downward molecular weight (MW) shift and tight banding pattern for both the ∼95 and ∼76 kDa bands on Western blot (Fig. 6*A*). At 9 to 24 h after cellular uptake, the ∼76 kDa form with processed glycans is the predominant species present (Fig. 6*A*). In contrast, the intermediate forms of oxidized rhGAA or rhGAA–AOAA maintained a smeared banding pattern indicative of gross heterogeneity presumably because of a lack of glycan processing, even after 24 h after cellular uptake (Fig. 6*A*).

To confirm whether the observed differences were indeed due to altered glycan processing, we incubated cell lysates harvested 24 h after internalization with peptide:N-glycanase F (PNGase F), which is an endoglycosidase that cleaves glycans between the innermost GlcNAc and asparagine residues to release N-glycans from protein backbone. As expected, PNGase F treatment resulted in identical, tight band patterns for the unmodified and the chemically modified glycoforms of rhGAA (Fig. 6*B*). Together, these results indicate that C7 modification of sialic acid in rhGAA-oxidized and rhGAA–AOAA blocks lysosomal sialidase activity and subsequent glycan trimming *in cellulo*.

### Effects of glycan trimming and proteolytic processing on rhGAA glycogen kinetics

Because lysosomal processing is known to increase the affinity of GAA for its natural substrate glycogen (10, 17), we next sought to use the modified rhGAAs to dissect the relative contributions of proteolytic processing and glycan trimming on GAA enzyme kinetics. We used *in cellulo* processed rhGAA–AOAA (fibroblast cell lysates derived from patients with Pompe disease after 16 h of rhGAA uptake and additional 24 h of processing) as a representative rhGAA sample that underwent proteolytic processing without complex N-glycan trimming. We utilized deglycosylated rhGAA–kif to represent an rhGAA sample that underwent glycan trimming *in vitro* without proteolytic processing. Importantly, when rhGAA–kif was treated with Endo H, it was completely deglycosylated at site 5, which is proximal to the active site (19) and thus most likely to impede substrate binding if decorated with large glycan structures.

**Figure 7:**
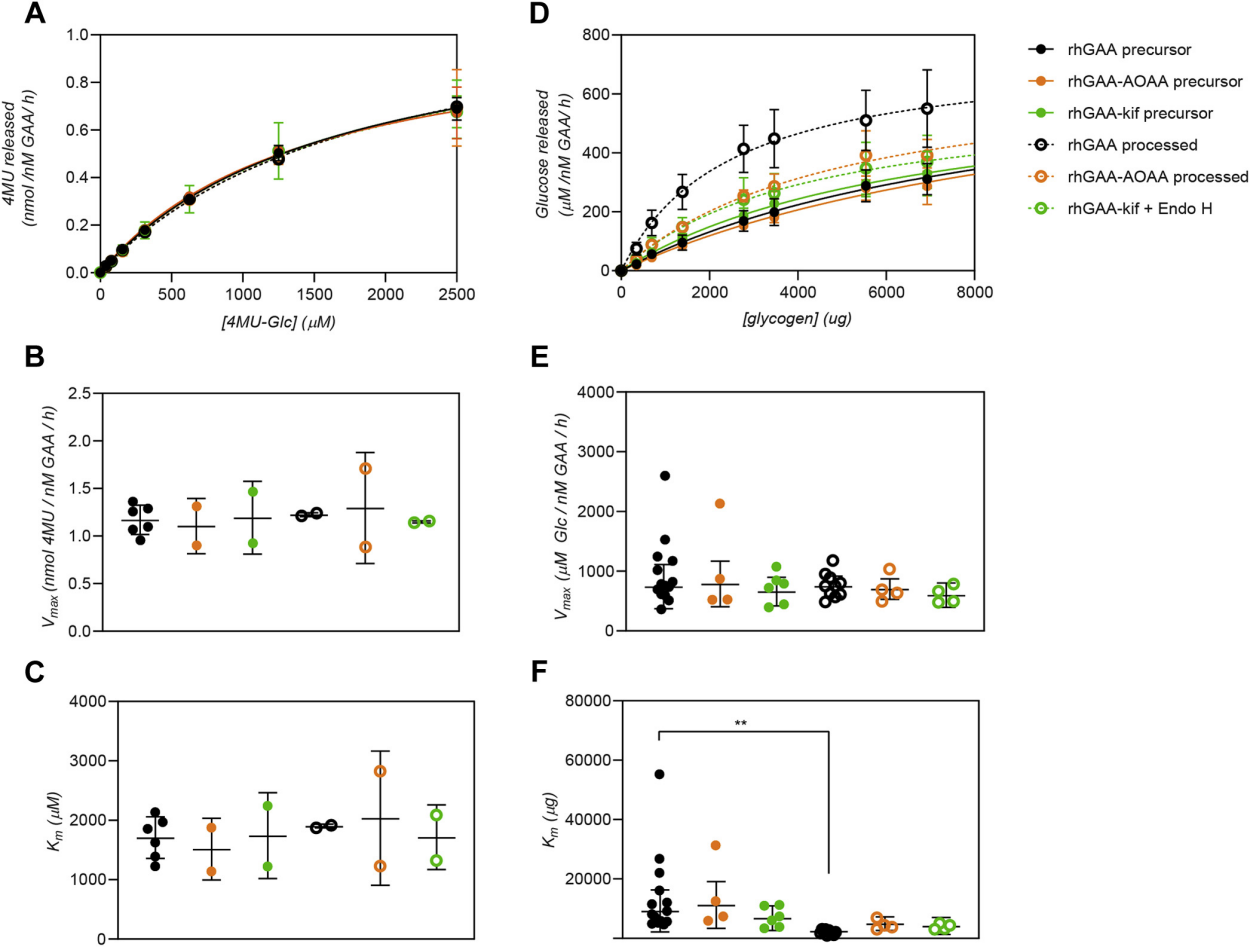
**Glycan trimming is necessary for rhGAA activation.** Michaelis–Menten kinetics of various rhGAA forms using 4MU-α-Glc or glycogen as substrates. Reactions were carried out for 60 min at 37 °C. Data points were fitted to the Michaelis–Menten equation using Prism (GraphPad), and error bars represent the mean ± SD. One-way ANOVA with Fisher’s LSD post hoc test was also performed using Prism (GraphPad). A *p*-value of <0.005 is denoted by two *asterisks* (∗∗). *A*, Michaelis–Menten kinetics of rhGAA forms using varying amounts of the small fluorogenic substrate 4MU-α-Glc. *B*, *V*_*max*_ for rhGAA forms in 4MU-α-Glc hydrolysis. *C*, *K*_*m*_ values for rhGAA form sin 4MU-α-Glc hydrolysis. *D*, Michaelis–Menten kinetics of rhGAA forms using varying amounts of bovine liver glycogen. *E*, *V*_*max*_ for rhGAA forms in glycogen hydrolysis. *F*, *K*_*m*_ values for rhGAA forms in glycogen hydrolysis. GAA, acid alpha-glucosidase; rhGAA, recombinant human GAA.

First, we assessed GAA enzyme kinetics on the synthetic fluorogenic small-molecule substrate 4-methylumbelliferyl α-D-glucopyranoside (4MU-Glc). All rhGAA samples including unmodified precursor rhGAA, *in cellulo* processed unmodified rhGAA, rhGAA–AOAA, *in cellulo* processed rhGAA–AOAA, rhGAA–kif, and deglycosylated rhGAA–kif displayed similar activity toward 4MU-Glc (Fig. 7, *A*–*C*, Table 1). These results confirm that none of the chemical modifications (including potential off-target amino acid oxidation) affected the catalytic potential of the modified rhGAAs toward the small-molecule substrate.

**Table 1:** Kinetics of 4MU-Glc hydrolysis

rhGAA form	*V*_*max*_ ± SD (nmol/nM GAA/h)	*K*_*m*_ ± SD (μM)	*V*_*max*_*/K*_*m*_
rhGAA precursor	1.2 ± 0.15	1709 ± 350	0.000684
rhGAA–AOAA precursor	1.1 ± 0.29	1516 ± 520	0.000729
rhGAA–kif precursor	1.2 ± 0.38	1741 ± 722	0.000685
rhGAA processed	1.2 ± 0.02	1903 ± 31	0.000643
rhGAA–AOAA processed	1.3 ± 0.58	2036 ± 1130	0.000636
rhGAA–kif + Endo H	1.1 ± 0.01	1715 ± 542	0.000669

We next examined the impact of glycan trimming on GAA activity toward the natural substrate, glycogen. As expected, unmodified rhGAAs exhibited an increase in catalytic efficiency after *in cellulo* processing, with a statistically significant decrease in *K*_*m*_ (*p* = 0.0032, one-way ANOVA with Fisher’s least significant difference (LSD) post hoc test; Fig. 7, *D*–*F*). While the increase in affinity was substantial (approx. 4-fold *K*_*m*_ decrease), it did not approach previously reported fold changes (10, 17). This could be because our assays were carried out at pH 4.8 (the average pH of lysosomes) rather than pH 4.0 to 4.5 (the pH optimum of GAA, which was used in prior studies) (10, 17). Differences in the purity of the mature rhGAA preparation, incomplete processing in the compromised endolysosomal pathway of cells of patients with Pompe disease (see Discussion), or differences in the macromolecular complexity of the glycogen substrate preparations could also have affected the *K*_*m*_ values obtained. Indeed, we observed substantial differences in the MW distributions for different commercial glycogen preparations (including different lots of the same catalog product) and variable kinetics on each of the different glycogen preparations (Fig. S9, Table S2). Nonetheless, these results show that the combined proteolytic processing and glycan trimming of rhGAA within the endolysosomal system had a significant, positive impact on glycogen hydrolysis.

Precursors rhGAA–AOAA and rhGAA–kif had an apparent affinity for glycogen that was comparable with unmodified rhGAAs (within the margin of error, *p* = 0.50 and 0.34, respectively; Table 2). After proteolytic processing, but not glycan trimming *in cellulo*, processed rhGAA–AOAA exhibited only an approximately 2-fold, statistically insignificant decrease in *K*_*m*_ (*p* = 0.15, Table 2) compared with precursor unmodified rhGAA. Similarly, rhGAA–kif that underwent near-complete deglycosylation, but not proteolysis (rhGAA-kif + Endo H), also displayed only partial activation with an approximately 2-fold, statistically insignificant decrease in *K*_*m*_ (*p* = 0.09). In both cases, the observed *K*_*m*_ for these processed modified rhGAAs were not statistically different from their respective precursor rhGAAs (rhGAA–AOAA precursor *versus* rhGAA–AOAA processed, *p* = 0.09; rhGAA–kif precursor *versus* rhGAA kif + Endo H, *p* = 0.43). Together, these results support a model wherein both proteolytic processing and glycan trimming independently contribute toward GAA activation, with both being necessary for complete rhGAA activation. The fact that these differences are only observed on a complex macromolecular structure (*i.e.*, glycogen, but not 4MU-Glc) suggests that both the retention of bulky glycan structures and incomplete proteolysis can hinder active site or secondary binding site accessibility and subsequent kinetics.

**Table 2:** Kinetics of glycogen hydrolysis

rhGAA form	*V*_*max*_ ± SD (μM/nM GAA/h)	*K*_*m*_ ± SD (μg)	*V*_*max*_*/K*_*m*_	*K*_*m*_ ± SD (~mg/ml)
rhGAA precursor	741 ± 370	9240 ± 7048	0.080	58 ± 44
rhGAA–AOAA precursor	787 ± 381	11,244 ± 7854	0.070	70 ± 49
rhGAA–kif precursor	658 ± 239	6822 ± 4165	0.097	43 ± 26
rhGAA processed	750 ± 168	2445 ± 1367	0.307	15 ± 9
rhGAA–AOAA processed	700 ± 172	4940 ± 2286	0.142	31 ± 14
rhGAA–kif + Endo H	599 ± 204	4199 ± 2866	0.143	26 ± 18

## Discussion

It has long been established that rhGAA undergoes proteolytic cleavage and glycan trimming events within the endolysosomal system after cellular uptake, resulting in increased glycogen hydrolytic activity (10, 17). However, owing to the concomitant nature of glycan trimming and proteolytic processing, it has been difficult to discern the relative contributions of each type of processing. Here, we generated modified rhGAAs that allowed us to distinguish, for the first time, the individual contributions of glycan trimming and proteolysis for enhancing rhGAA hydrolytic activity toward its natural substrate glycogen.

We chemically modified terminal sialic acids on complex-type N-glycans on rhGAA and found that modification at the C7 position by oxidation or by AOAA conjugation with a non-native oxime bond rendered sialic acids nonhydrolyzable *in vitro* and *in cellulo*. Because glycan trimming proceeds in a sequential, “outside-in” manner *via* resident lysosomal exoglycosidases, this blockade of sialidase activity rendered complex sialylated N-glycans entirely nonhydrolyzable. While we focused on the C7 position of sialic acid, it is likely that modifications to other exposed terminal sugars such as galactose would have a similar effect, as lysosomal exoglycosidases typically have precise requirements for substrate recognition (20, 29, 37, 38). Likewise, similar inhibition of glycan processing could be imparted by other chemistries that introduce unnatural linkages at critical enzyme recognition sites on terminal sugars. Conjugation strategies that have been widely utilized for engineering of nonenzymatic proteins (*e.g.*, antibody–drug conjugates) may thus impact postdelivery glycan trimming when utilized in this context, limiting utility for the development of improved ERTs.

When delivered to cultured fibroblasts, our C7-modified rhGAAs underwent proteolysis comparable to that observed for unmodified rhGAA, demonstrating that proteolysis is not dependent on glycan trimming. More importantly, the glycogen hydrolytic activity of these proteolytically processed rhGAAs with intact glycans trended toward maturation-associated increases, but the increases were far less than those observed for unmodified rhGAA after *in cellulo* processing. These results demonstrate, for the first time, that glycan trimming is an essential step for attaining maturation-associated increases in GAA glycogen hydrolytic activity.

We also generated a modified rhGAA, rhGAA–kif, that could undergo nearly complete glycan trimming *in vitro* by treatment with Endo H in the absence of any proteolytic processing. Importantly, LC-MS verified that Endo H treatment of rhGAA–kif completely removed bulky glycan structures at N-glycan site 5, the site most proximal to the active site of the enzyme, although some complex glycan structures were retained at the more distal sites 6 and 7. Glycan trimming with Endo H resulted in trends toward increased glycogen hydrolytic activity, again to a lesser extent than that observed for unmodified *in cellulo*–processed rhGAA. These results suggest that glycan removal alone can increase GAA activity, albeit only partially. Because the complex glycans at sites 6 and 7 were refractory to Endo H treatment, it is also possible that removal of these glycans would result in further activity increases through mechanisms separate from direct steric hindrance to the GAA active site, as these sites are distal to the active site.

It has previously been shown that endolysosomal processing of rhGAA dramatically reduces the *K*_*m*_ for glycogen, but not the *K*_*m*_ for low-molecular-weight substrates (10, 17). The GAA substrate-binding pocket is relatively narrow and is buried within several beta strands within the catalytic domain (19), suggesting a significant potential for steric hindrance with large substrates after the addition or retention of moieties that are not naturally present on the mature enzyme, as we observed when glycan trimming was blocked. In addition, rhGAA contains a secondary glycogen substrate-binding site on the same face as the active site that has been proposed to contribute to processivity (19). This secondary site becomes accessible after *in vitro* proteolysis, and is notably free of glycans, suggesting that effects of lysosomal proteolysis on kinetics could be mediated largely here (19). In both cases, the structure of rhGAA demonstrates how endogenous kinetics may not be appropriately modeled when measuring activity on small analogues, as observed here and in prior studies (25, 39, 40).

In this study, we observed smaller maturation-associated increases in glycogen kinetics than previously reported after *in cellulo* processing (10, 17). One likely explanation for this is that rhGAA maturation was inefficient or incomplete in our cell lysates of patients with Pompe disease, in contrast to previously published results obtained using purified mature GAA from WT production cell lines or human placenta. The endolysosomal pathway exhibits a range of abnormalities in Pompe cells, including aberrant trafficking and buildup of endocytic and autophagic vesicles (41), abnormal CI-MPR trafficking (16), reduced acidification of endosomes and lysosomes (41), and potentially reduced levels of resident glycosidases and proteases. Because rhGAA is internalized and processed through the endolysosomal pathway in a CI-MPR–dependent and pH-dependent manner, it is likely that such defects interrupt normal rhGAA trafficking and maturation. In this context, these cellular defects associated with Pompe disease are highly clinically relevant. These cellular defects have been proposed to underlie the resistance of Pompe patient Type II myofibers to ERT (41), and, when considered alongside the present results, highlight the need to develop rhGAA ERTs that are very efficiently delivered as they are processed in a compromised endolysosomal environment.

Only a small fraction of intravenously delivered rhGAA reaches the interstitial space in target tissues such as skeletal muscle (13). Because first-generation ERTs contain only low levels of M6P, these rhGAAs have poor affinity for the CI-MPR and thus limited cellular uptake and lysosomal targeting (13, 24, 39). This inefficiency is believed to underlie the suboptimal clinical results that have been obtained with first-generation ERTs. Long-term results with these therapies (*i.e.*, Myozyme and Lumizyme) have shown that, although they can decrease mortality, prevent deterioration of respiratory function, and increase ambulatory capacity, results are highly variable. Significant morbidity may be present even after treatment, particularly in patients in whom treatment is initiated later in the disease course (42). Even infantile-onset patients who begin ERT very early in life typically develop progressive myopathy resulting in significant disability (43, 44, 45).

Thus, development of next-generation therapies for Pompe disease has focused on optimizing enzyme delivery to targeted muscle cells. Several strategies have been shown to be effective. A novel production cell line coupled with novel manufacturing processes have been used to produce rhGAA with an increased M6P content, in particular with a high percentage of bisphosphorylated glycans, and this ERT product exhibits improved uptake *in vitro* and enhanced glycogen clearance *in vivo* (46). Other approaches have utilized glycosylation-independent lysosomal targeting with chimeric fusion proteins (47) or chemical conjugation of synthetic M6P moieties onto amino acids or glycans on rhGAA (25). Although efforts such as these have made considerable progress toward producing an rhGAA ERT with improved muscle targeting, the present results demonstrate the importance of postdelivery processing as an essential, yet a largely underappreciated aspect for developing an effective treatment for Pompe. The results herein demonstrate the requirement of both proteolytic processing and glycan trimming to fully activate GAA. Chemical modifications to terminal sugar residues appear to introduce unintended impediments to the GAA maturation process and limit GAA hydrolytic activity toward its natural substrate, glycogen.

The effectiveness of rhGAA ERT is further complicated by widespread cellular pathology that is already present when treatment is initiated. This disease state renders affected cells refractory to ERT because of decreased surface abundance of the CI-MPR (16), disrupted endocytic trafficking (41), and alkalinated endosomes and lysosomes (41). Our results suggest that these cellular defects impede not only cellular uptake of ERT but also rhGAA processing and activation after internalization, potentially reducing the effectiveness of ERTs. It is therefore critical to develop next-generation rhGAA ERTs that are efficiently internalized in target muscle cells and capable of being fully processed and activated to maximize the therapeutic benefits of ERT in the face of all the aforementioned challenges.

Even with an ideal rhGAA ERT that is targeted and processed efficiently, the presence of extensive pre-existing cellular damage will likely result in an extended timeframe for cellular and clinical benefits to appear (48). Lysosomal clearance of accumulated substrate will likely proceed slowly at first until the storage burden is reduced to levels that no longer obstruct endolysosomal function. Subsequent ERT could then have even greater effectiveness leading to more pronounced clinical benefits for patients. Recent studies with next-generation rhGAA ERTs show that efficient muscle targeting and proper cellular processing leads to reversal of muscle pathology and restores a number of affected cellular pathways (47, 48), thus providing a glimmer of hope that a much more effective ERT can be developed for Pompe.

## Experimental procedures

### Cell lines and culture maintenance

A fibroblast cell line (GM12932) from a patient with Pompe disease was obtained from Coriell Institute and grown in Dulbecco's modified Eagle's medium (DMEM) (Gibco, 200 mM L-glutamine, 4.5 g/l D-glucose, 110 mg/l sodium pyruvate) supplemented with 15% fetal bovine serum (FBS) and maintained at 37 °C with 5% CO_2_. HEK293F cells were purchased from Thermo and maintained in suspension in FreeStyle 293 media (Gibco) at 37 °C, 125 rpm, and 5% CO_2_. A CHO-S cell line stably overexpressing rhGAA (previously generated at Amicus Therapeutics) was maintained in suspension in CD OptiCHO medium (Gibco) supplemented with 1x GlutaMAX at 37 °C, 125 rpm, and 5% CO_2_.

### Chemical modification of rhGAA

A clinical batch of rhGAA (Lumizyme) was obtained from Sanofi Genzyme. Lumizyme is produced in Chinese hamster ovary (CHO) cells. AOAA was obtained from Cayman Chemical company. rhGAA was the buffer exchanged with 30 volumes of 0.1 M sodium acetate (pH 5.6) by centrifugal filtration using a 10-kDa molecular weight cutoff (MWCO) spin concentrator at 4 °C. A final concentration of 10 mM sodium metaperiodate (from a 200 mM stock solution made in deionized water and stored in the dark) was added to 1 ml of a 5 mg/ml sample of buffer-exchanged rhGAA and incubated on ice for 30 min. Excess sodium metaperiodate was consumed by the addition of 0.5 ml of 50% glycerol (made in 0.1 M sodium acetate, pH 5.6) and further incubation on ice for 15 min. Oxidized rhGAA was buffer-exchanged again as above and combined with a final concentration of 5.5 mM of AOAA (from ∼20 mM stock solutions made in 0.1 M sodium acetate, pH 5.6) in the presence of 0.1 M aniline used as a catalyst and incubated shaking at 300 rpm for 4.5 h at 37 °C. Chemically modified rhGAAs were subjected to buffer exchange, snap frozen at a concentration of 5 mg/ml, and stored at −80 °C.

### Fluorescence-based activity assay of GAA

Enzymatic activity of rhGAA was assayed using the synthetic fluorescent substrate 4-methylumbelliferyl α-D-glucosaminide (4MU-Glc, Sigma). Reaction mixtures (50 μl) contained <10 nM enzyme or cell lysates in 0.1 M sodium acetate (pH 4.8) and a final concentration of 1 mM 4MU-Glc. Reactions were performed for 1 h at 37 °C and stopped by the addition of 125 μl of 1 M glycine–NaOH (pH 10.3). Fluorescence of released 4MU was measured using a SpectraMax iD5 plate reader, with excitation and emission wavelengths of 360 and 460 nm, respectively. Reactions were set up in duplicates (with an n = 6 for unmodified precursor rhGAA alone), and measurements were corrected for background emission from reactions containing no enzyme. For all assays performed, substrate turnover was linear and under 10% for the duration of the assay. Data were analyzed and graphed with Prism (GraphPad).

### Sialic acid estimation using HPAEC–PAD

To determine the sialic acid content in GAA/modified GAA, 5 μg samples in duplicate were hydrolyzed with 0.05 N TFA at 80 °C for 60 min, dried in a SpeedVac and reconstituted in HPLC water. 10 μl of the reconstituted sample was loaded on a Dionex CarboPac PA10, 2 × 250 mm analytical column for analysis by HPAEC-PAD. The eluent composed of Mobile Phase A [100 mM sodium hydroxide (NaOH) in MilliQ water] and mobile phase B [100 mM NaOH + 1 M sodium acetate (NaOAc) in MilliQ water] was set at a total flow rate of 0.250 ml/min. The mobile phases were used in a gradient profile of mobile phase A from 7% to 30% for 11 min and then from 30% to 7% in 1 min. The analytical column was re-equilibrated with 7% mobile phase A for 15 min before the next injection. The total run time was 27 min. Sialic acid expressed as moles of sialic acid per mole protein was quantified based on peak area *versus* nominal concentration of an eight-point calibration standard curve of sialic acid.

### Glycopeptide analysis by MS

About 17.5 to 25 μg of unmodified and modified rhGAA were denatured with urea, reduced with dithiothreitol, alkylated with iodoacetamide, and digested with trypsin and Lys-C in a 1:10 ratio of protease to protein. The released glycopeptides were desalted by solid-phase extraction on a microspin C18 column, dried in a SpeedVac, and reconstituted with 0.2% formic acid in 2% acetonitrile in LC-MS grade water. Five micrograms of the purified glycopeptides were loaded on a Waters Acquity 1 × 50 mm CSH C18 UPLC analytical column for liquid chromatographic separation and high-resolution tandem MS analysis. The LC apparatus consisted of a NanoLC system operated at a flow rate of 35 μl/min. The eluent was composed of mobile phase A (0.1% formic acid in LC-MS grade water) and mobile phase B (0.1% formic acid in LC-MS grade acetonitrile). Mobile phase B was used in a gradient profile of 1% to 50% for 90 min to separate the glycopeptides and then up to 95% mobile phase B for 15 min to elute other highly retentive peptides off the column. Finally, the column was re-equilibrated with 99% of mobile phase A and 1% of mobile phase B for the next injection. The MS analysis was carried out by positive electrospray ionization mode on an Orbitrap Fusion Lumos Tribrid operated at a resolution of 120,000 at *m/z* = 120. For the MS1 level data generation, mass isolation was performed in the quadrupole in the scan range of 300 to 2000 *m/z*. The automatic gain control target was set at 2.0e5 with a maximum injection time of 50 ms. At the MS2 level, data were acquired by data-dependent MS2 OT HCD at a resolution of 15,000 at *m/z* = 120. MS raw data were acquired using Xcalibur. The raw data were analyzed by the peak list–generating software Genedata Expressionist (version 14.0.1). For the search engine, we utilized the peptide mapping search algorithm within Genedata Expressionist (version 14.0.1). The parameters used in the raw data searches are defined as follows: The raw spectra were searched against the *Homo sapiens* (human) GAA protein sequence (UniProtKB # P10253). The final glycopeptide data were reported based on zero missed cleavages from Trypsin and Lys-C proteases (which cleave at the C-terminal side of Lys and Arg residues). The full MS precursor mass tolerance was set to 10 ppm, and MS2 peptide fragment mass tolerance was set to 40 ppm. Fixed modifications included carbamidomethyl (Cys). Variable modifications included modified glycans (Asn), oxidation (Met), Gln->pyro-Glu (N-term Gln). A total of three fixed or variable modifications were allowed per peptide. Allowed N-linked glycan modifications (modified at Asn residues in sequons consisting of Asn-X-Ser/Thr, where X is any amino acid except proline) were included in a user-defined library that consisted of oligomannose, hybrid, and biantennary complex-type N-glycans that were previously reported for human GAA (11, 13, 15, 26). In these peptide mass fingerprint experiments, Genedata Expressionist (version 14.0.1) was used for the preliminary identification of glycopeptide spectra with a minimum threshold score of 2, which was then manually validated. Glycopeptides were identified by the presence of unique glycan oxonium ions in the HCD spectra and high-resolution accurate mass measurements in the MS1 spectra at a mass accuracy of 10 ppm or less (Supplemental Tables).

### Purification of recombinant human neuraminidases NEU1 and NEU4

Plasmids encoding FLAG-tagged human NEU1 and NEU4 were purchased from OriGene. DNA were transfected into HEK293F cells in suspension culture using a 1:3 ratio of DNA to polyethyleneimine (1 mg/ml). An equal volume of fresh culture media supplemented with 2.2 mM valproic acid was added 16 to 18 h after transfection, and the cells were allowed to grow for 2 to 3 additional days and then harvested. Cells were lysed with 1x tris buffered saline (TBS), pH 7.4, supplemented with 1x EDTA-free Halt protease inhibitors (Thermo) and 0.1% Triton X-100. FLAG-tagged NEU1 and NEU4 were purified from whole-cell lysates using FLAG agarose (Sigma). Eluates obtained from using 0.15 mg/ml 3 x FLAG peptide (AnaSpec) dissolved in 1x TBS, pH 7.4, were concentrated using a 10-kDa MWCO spin concentrator and diafiltrated to remove FLAG peptide. Concentrated proteins (NEU1-1.99 mg/ml, NEU4-2.3 mg/ml) were quantified using the Bradford assay and stored at 4 °C and used within a month or flash-frozen and stored at −80 °C for extended durations.

### Neuraminidase treatment of rhGAA

Bacterial neuraminidase from *Arthrobacter ureafaciens* recombinantly expressed in *Escherichia coli* (5 units/ml) was obtained from Sigma. 20 μg of each rhGAA sample was treated with 5 μl of a 1:100 diluted (in TBS) solution of bacterial neuraminidase for 20 h at 37 °C. The final pH of the reactions was adjusted to 4.8 with addition of 0.1 M sodium acetate (pH 4.8), and final reaction volumes were 40 μl. Untreated samples were subjected to the same conditions, except TBS was added instead of an exoglycosidase. 2.5 μg of treated rhGAA was denatured using NuPAGE SDS sample buffer (Thermo) containing a reducing agent and set aside for Eastern/Western blotting, while the remainder of the samples (containing 17.5 μg rhGAA) were flash-frozen and stored at −80 °C before denaturing with urea for MS analysis. NEU1 and NEU4 treatments were performed on 2 μg rhGAA by adding 10 μl of NEU1/NEU4 (concentrations of both were ∼2 mg/ml) or 2 μl 1:100 diluted (in TBS) bacterial neuraminidase in a final volume of 40 μl and a pH of 4.8, obtained by adding a final concentration of 0.1 M sodium acetate (pH 4.8) to the reaction mixture.

### Cell uptake and GAA processing

To assess GAA proteolytic and glycan processing, fibroblasts of patients with Pompe disease were treated with 500 nM rhGAA or 500 nM modified rhGAAs for 16 to 18 h. Briefly, 200,000 (6-well) or 500,000 (10 cm plate) cells were seeded in growth media (DMEM, 4.5 g/l D-glucose, L-glutamine, 110 mg/l sodium pyruvate, 15% FBS) and allowed to adhere overnight. Growth media were then aspirated and replaced with uptake media (Ham’s F-10, 10% heat-inactivated FBS, 200 mM L-glutamine, 3 mM Pipes-HCl, pH 6.8) containing 500 nM of rhGAA or modified rhGAA, and the cells were allowed to internalize rhGAA for 16 to 18 h at 37 °C. After uptake, cells were treated with 1.5 M Tris HCl (not Tris base, made from powder without adjusting pH) for 15 min and then with 1 M sodium phosphate monobasic pH 4.0. Cells were then washed with PBS, harvested, and stored at −80 °C or lysed immediately in 0.1 M sodium acetate (pH 5.6) supplemented with 1x EDTA-free HALT protease inhibitors (Thermo) and 0.1% Triton X-100. Cell debris were pelleted, and supernatants were used for total protein quantification by Bradford assay followed by Western blots and 4MU-Glc activity assays. For time point experiments, cells were washed with 1X PBS, pH 7.4, after a 16 to 18 h uptake period and incubated with growth media until they were harvested and processed at designated time points.

### PNGase F digestion

PNGase F kit (P0708) was obtained from New England Biolabs and glycoproteins from the cell uptake lysates were denatured before release of N-glycans following the manufacturer’s directions. Briefly, 15 ug of total protein from each lysate was denatured in duplicate using a final concentration of 1X glycoprotein-denaturing buffer at 100 °C for 10 min. Denatured glycoproteins were then cooled for 10 min and treated with either 1 μl of PNGase F or PBS as a control in GlycoBuffer and NP-40 mix for 4 h at 37 °C. Proteins were then analyzed by Western blotting as described below.

### Western and Eastern blots

After quantification by the Bradford assay, equal amounts of total protein were boiled in NuPAGE SDS sample buffer (Thermo) containing a reducing agent. Three micrograms of whole-cell lysates and 10 ng of unprocessed enzymes were resolved on a 4 to 12% Bis-Tris Midi Gel (Thermo). Proteins were transferred to a nitrocellulose membrane using the iBlot 2 dry blotting system (Thermo). Membranes were blocked with 3% bovine serum albumin (BSA) in PBS with 0.1% Tween 20 for 30 min at room temperature (RT). After incubation, blots were probed with rabbit anti-human GAA (1:1000, 1 h, RT; ab137068, Abcam) or rabbit anti-actin (1:2500, 1 h, RT; ab179467, Abcam) and then probed with donkey anti-rabbit secondary antibody conjugated with IRDye 680 or 800 (1:5000–1:10,000, 1 h, RT; LI-COR) for detection. Odyssey CLx (LI-COR) was used for scanning blots. For Eastern/lectin blots, 0.5 to 1 μg of purified proteins were resolved on a 4 to 12% Bis-Tris mini gel (Thermo) and blotted as noted above. Membranes were blocked in 5% BSA in TBS-0.1% Tween 20 supplemented with 1 mM MnCl_2_ and CaCl_2_ for at least 1 h and incubated with 100 μg of biotinylated WGA (Vector Laboratories) diluted in the blocking buffer overnight at 4 °C. Blots were probed with streptavidin conjugated to IRDye 800 (1:10,000, 1 h, RT; LI-COR) and also probed for GAA as noted above.

### Purification of rhGAA containing primarily high-mannose-type N-glycans

A CHO-S cell line stably overexpressing rhGAA was grown in the presence of 10 μM kif, a potent alpha-mannosidase inhibitor, in a medium containing 50% CD OptiCHO (Gibco) and 50% HyCell (Cytiva) supplemented with 4 mM glutamine, 2 g/l glucose, 13 mM NaHCO_3_, and 1.5 g/l Pluronic F68 (Spectrum) and maintained as a batch re-feed culture for 14 days at 37 °C, 125 rpm, and 5% CO_2_. On days 6, 10, and 14, half of the culture was harvested through centrifugation at 3000*g* for 15 min, filtered through a 0.22-μm filter, and stored at −80 °C. The remaining culture was refreshed to the original volume with a fresh medium supplemented with 10 μM kif. Supernatants were loaded at 13 ml/min onto a 50-ml Q Sepharose fast-flow XK-26 Anion Exchange Chromatography column (Cytiva) equilibrated with 40 mM sodium phosphate, pH 7.1. After sample loading, the column was washed with five column volumes of 40 mM sodium phosphate (pH 7.1) and then five column volumes of 40 mM sodium phosphate (pH 6.3). rhGAA–kif was eluted with an isocratic elution containing 180 mM sodium chloride in 40 mM sodium phosphate (pH 6.3), and fractions were collected. Fractions enriched with precursor rhGAA–kif (110 kDa) (analyzed by SDS-PAGE and Western blotting) were pooled and dialyzed into 25 mM Tris HCl (pH 6.5) overnight and adjusted to contain 1 M ammonium sulfate before further purification using Hydrophobic interaction chromatography. The sample was loaded at 13 ml/min onto a 25 ml Phenyl Sepharose 6 Fast-Flow High Sub XK-26 Hydrophobic Interaction Chromatography column (Cytiva) equilibrated in 25 mM Tris HCl, 1 M ammonium sulfate (pH 6.5). After loading, the column was washed with 25 mM Tris HCl and 1 M ammonium sulfate (pH 6.5) for five column volumes, and then 25 mM Tris HCl and 0.5 M ammonium sulfate (pH 6.5) for seven column volumes, and then 25 mM Tris HCl and 0.3 M ammonium sulfate (pH 6.5) for five column volumes. The rhGAA–kif was eluted with an isocratic elution containing 0 M ammonium sulfate in 25 mM Tris HCl (pH 6.5) for 10 column volumes. Fractions enriched with precursor rhGAA–kif were pooled and dialyzed into 100 mM sodium acetate, 250 mM sodium chloride (pH 5.0) overnight and then concentrated using a Centricon Plus 70 centrifugal filter unit with a 30 kDa MWCO. The sample was further purified using pseudo-affinity chromatography using a Superdex 200 pg 26/600 column (Cytiva) (traditionally used for size-exclusion chromatography) equilibrated with 100 mM sodium acetate and 250 mM sodium chloride (pH 5.0) and eluted isocratically using the same buffer as the mobile phase. rhGAA is retarded on the column because of its affinity for the dextran backbone of the Superdex matrix (18). In addition, precursor rhGAA has a lower affinity for the Superdex matrix than processed forms, causing it to elute before processed forms. Fractions containing >95% pure precursor rhGAA were pooled, concentrated, and stored frozen at −80 °C until further use.

### Endo H treatment of unmodified rhGAA and rhGAA–kif

Endo H (500,000 units/ml) was obtained from New England Biolabs and used as per the manufacturer’s instructions for nondenaturing conditions. 60 μg of rhGAA or rhGAA–kif were incubated with Endo H for a total of 10 days at 30 °C in a thermomixer set to shake at 300 rpm. The treatment was initiated by adding 12,000 U of Endo H in a final reaction volume of 60 μl. The reaction mixture was supplemented with additional Endo H (9000 U) every 3 days until the reaction volume reached 100 μl on day 10. Untreated controls were included, and proteins were assayed for 4MU-Glc hydrolysis activity at the end of the treatment period to ensure retention of enzymatic activity. Proteins were then flash-frozen in aliquots at −80 °C until further use.

### Size-exclusion chromatography, phenol sulfuric acid assay of glycogen, and calculation of the weighted average MW of glycogen

Bovine liver glycogen (Sigma G088, lot # SLBF8199V and SLBZ0559) and rabbit liver glycogen (Sigma G8876, lot #SLBX9591) were characterized in this study, and bovine liver glycogen (Sigma G088, lot # SLBZ0559) was used for kinetics. 1 ml of samples of glycogen at 125 mg/ml were subjected to size-exclusion chromatography using a Superose 6 16/600 prep grade column (Cytiva) and 0.1 M sodium acetate (pH 4.8) as the elution buffer. Fractions were collected, and glycogen in each fraction was detected by phenol sulfuric acid assay. 25 μl of each fraction diluted 1:100 in the elution buffer was added to wells of a clear, 96-well, polystyrene plate containing 25 μl of freshly prepared 5% phenol in water. After mixing, 125 μl of concentrated sulfuric acid was quickly added to each well using a manual multichannel pipette. The reaction was allowed to proceed for 5 min at RT before reading absorbance at A490 with a SpectraMax iD5 plate reader (49). The weighted average of the MW of all glycogen samples was calculated as follows: all glycogen assay values were divided by the maximum value in the set to obtain a relative glycogen abundance ratio for each fraction. The estimated MW for each fraction was multiplied by this ratio to obtain weighted MW. To calculate the weighted average, the weighted MW were summed and divided by the total sum of all glycogen abundance ratios. Values outside of the peak were excluded.

### Estimation of glycogen concentration by HPAEC-PAD

Three-microliter aliquots in triplicate were pipetted in 0.5-ml Safe-Lock microcentrifuge tubes and dried in a SpeedVac for 30 min. Two replicates were then hydrolyzed in 150 μl of 4 M TFA in water at 100 °C for 4 h and dried in a SpeedVac. A nonhydrolyzed replicate was used for the determination of glucose background. Dried hydrolysates and nonhydrolyzed sample were resuspended in 150 μl of degassed MilliQ water, and 10 μl was loaded on a Dionex CarboPac PA10 2 × 250 mm analytical column for analysis by HPAEC-PAD. The eluent composed of mobile phase A [100 mM sodium hydroxide (NaOH) in MilliQ water] and mobile phase B [100 mM NaOH + 1 M sodium acetate (NaOAc) in MilliQ water] was set at a total flow rate of 0.250 ml/min. The mobile phases were used in an isocratic profile of mobile phase A (98%) and mobile phase B (2%). The total run time was 7 min. Released glucose expressed in nmol/ml was identified by retention time and quantified based on an eight-point calibration standard curve of glucose. The conversion factor of 1 mg/ml glycogen equivalent to 6173 nmol/ml free glucose was used to convert mole of glucose to glycogen amount.

### *In vitro* glycogen hydrolysis by GAA

A final concentration of 0.3 to 0.5 nM of rhGAA (unprocessed) and lysates (processed GAA) from uptake assays were incubated with varying concentrations of glycogen in a final volume of 150 to 190 μl for 60 min at 37 °C. The assay buffer was composed of 0.1 M sodium acetate and 0.5 mg/ml BSA adjusted to pH 4.8. The reaction was stopped by boiling at 95 °C for 5 min and then stored at 4 °C until analyzed by HPAEC-PAD. A volume of lysates containing an equivalent specific activity of GAA (determined by 4MU-Glc hydrolysis as described above) as 0.3 to 0.5 nM of unprocessed rhGAA was used for glycogen hydrolysis.

### Glycogen kinetics determined by HPAEC-PAD

A Dionex ICS (Thermo Scientific) was used to measure glucose released from glycogen. After glycogen hydrolysis, 10 μl samples were injected for analysis. Each sample was injected twice. An isocratic 10-min method with the high-pH mobile phase consisting of 98% buffer B (100 mM NaOH, pH 13.0) and 2% buffer D (1 M sodium acetate, 100 mM NaOH) was used along with a Dionex CarboPac PA10 (2 × 250 mm) anion-exchange column. A fresh glucose standard curve was prepared by serially diluting glucose (Sigma # G6918; 100 ml) to obtain concentrations between 4000 μM and 0 μM in 0.1 M sodium acetate (pH 4.8) supplemented with 0.5 mg/ml BSA. Glucose peak areas were manually integrated, and glucose concentrations were interpolated from the standard curve. Nonlinear regression analysis using the Michaelis–Menten equation were performed on Prism (GraphPad). One-way ANOVA with Fisher’s LSD as the post hoc test was performed using Prism (GraphPad) with an n = 7 (14 total measurements on the Dionex ICS) for precursor rhGAA, an n = 5 (10 total measurements) for processed rhGAA, an n = 3 (6 total measurements) for rhGAA–kif, and an n = 2 (4 total measurements) for all other samples.

## Data availability

This article contains supporting information (Figs. S1–S8 and Tables S1 and S2). The raw mass spectrometry proteomics data have been deposited to the ProteomeXchange Consortium (http://proteomecentral.proteomexchange.org) *via* the PRIDE partner repository (50) with the dataset identifier PXD025285. Any data that support the findings of this study but not presented here are available from the corresponding author (Hung V. Do) or the first author (Nithya Selvan) upon reasonable request.

## Supporting information

This article contains supporting information.

## Conflict of interest

All authors, except J. B., are employees of Amicus Therapeutics Inc and hold equity in the company in the form of stock-based compensation.
