# Supplementary material for: Endolysosomal N-glycan processing is critical to attain the most active form of the enzyme acid alpha-glucosidase
Source: J Biol Chem. 2021 May 8;296:100769. doi: 10.1016/j.jbc.2021.100769 (PMC8191302; doi:10.1016/j.jbc.2021.100769)
Supplement: Supplemental Figures S1–S9 and Tables S1–S2 [file mmc8.docx]

**Endolysosomal processing of N-glycans on acid alpha-glucosidase is critical to attain the most active enzyme for hydrolyzing glycogen**

Nithya Selvan^1^, Nickita Mehta^1^, Suresh Venkateswaran^1^, Nastry Brignol^1^, Matthew Graziano^1^, M. Osman Sheikh^1^, Yuliya McAnany^1^, Finn Hung^1^, Matthew Madrid^1^, Renee Krampetz^1^, Nicholas Siano^1^, Anuj Mehta^1^, Jon Brudvig^2^, Russell Gotschall^1#^, Jill M. Weimer^1^, Hung V. Do^1*^

**Supporting information**

Figures S1-S9

Tables S1-S2

******Figure S1: Glycans observed at each of 7 N-glycan sites on unmodified rhGAA by LC-MS.** The schematic shows the most abundant glycan structure found on each site and the tables below the schematic show the relative abundance (%) of all other glycan structures found on each site. MS raw data were acquired using Xcalibur. Genedata Expressionist was used to process the raw data for glycopeptide identification. Glycan annotations are represented by symbols according to SNFG conventions in the schematic and Oxford notations in the tables. Site 7 in rhGAA is only occupied by glycan structures at a rate of ~50%.

**Figure S2: Glycans observed at each of 7 N-glycan sites on oxidized rhGAA by LC-MS.** The schematic shows the most abundant glycan structure found on each site and the tables below the schematic show the relative abundance (%) of all other glycan structures found on each site. MS raw data were acquired using Xcalibur and Genedata Expressionist was used to process the raw data for glycopeptide identification. Glycan annotations are represented by symbols according to SNFG conventions in the schematic and Oxford notations in the tables. “ox” denotes C7 oxidation of sialic acid. Site 7 in rhGAA is only occupied by glycan structures at a rate of ~50%.

**Figure S3: Glycans observed at each of 7 N-glycan sites on rhGAA-AOAA by LC-MS.** The schematic shows the most abundant glycan structure found on each site and the tables below the schematic show the relative abundance (%) of all other glycan structures found on each site. MS raw data were acquired using Xcalibur and Genedata Expressionist was used to process the raw data for glycopeptide identification. Glycan annotations are represented by symbols according to SNFG conventions in the schematic and Oxford notations in the tables. “ox” denotes C7 oxidation of sialic acid and blue line ( **|** ) denotes addition of AOAA to C7 oxidized sialic acid. Site 7 in rhGAA is only occupied by glycan structures at a rate of ~50%.

**Table S1: Total sialic acid (Neu5Ac) monosugar tabulation extrapolated from site specific N-glycan %RA obtained by LC-MS analysis for rhGAA and rhGAA-AOAA.** ~8.6 mol of sialic acid is present per mol of unmodified rhGAA. Treatment with 10 mM sodium metaperiodate results in ~8.5 mol C7-oxidized sialic acid per mol rhGAA, which translates to ~98% oxidation efficiency. ~4.1 mol AOAA are present per mol of protein in rhGAA-AOAA. The difference in total mol of Neu5Ac present per mol protein in unmodified rhGAA and rhGAA-AOAA is likely due to glycan microheterogeneity or may be a sampling artefact compounded by high resolution analysis.

|  | **Unmodified rhGAA (mol)** | **rhGAA-AOAA (mol)** |
| --- | --- | --- |
| **NeuAc** | 8.6 | 0.2 |
| **NeuAc Oxidized** | N/A | 4.4 |
| **NeuAc AOAA** | N/A | 4.1 |
| **Total** | 8.6 | 8.7 |

**Figure S4: Glycans observed at each of 7 N-glycan sites on rhGAA-kif by LC-MS.** The schematics show the most abundant glycan structure found on each site in rhGAA and rhGAA-kif and the tables show the relative abundance (%) of all other glycan structures found on sites 6 and 7 in rhGAA-kif. The relative abundance of glycan structures found on sites 1-5 are not shown since only high mannose type N-glycan structures were observed on these sites for rhGAA-kif. MS raw data were acquired using Xcalibur. Genedata Expressionist was used to process the raw data for glycopeptide identification. Glycan annotations are represented by symbols according to SNFG conventions in the schematic and Oxford notations in the tables. Despite culturing cells in the presence of 10 µM kifunensine, a potent mannosidase inhibitor, sites 6 and 7 of rhGAA-kif still had complex N-glycan structures. Site 7 in rhGAA is only occupied by glycan structures at a rate of ~50%. NF denotes glycopeptides that were ‘not found’ likely due to insufficient ionization.

**Figure S5: Glycans observed at each of 7 N-glycan sites on Endo H treated rhGAA-kif by LC-MS.** The schematics show the most abundant glycan structure found on each site in rhGAA-kif and Endo H treated rhGAA-kif and the tables show the relative abundance (%) of all other glycan structures found on sites 6 and 7 in Endo H treated rhGAA-kif. MS raw data were acquired using Xcalibur. Genedata Expressionist was used to process the raw data for glycopeptide identification. Glycan annotations are represented by symbols according to SNFG conventions in the schematic and Oxford notations in the tables. Endo H treatment resulted in only GlcNAc being detected at sites 1-5. GlcNAc was also the most abundant structure on sites 6 and 7 post Endo H treatment of rhGAA-kif, though these sites still contained a smaller percentage of complex structures. Site 7 in rhGAA is only occupied by glycan structures at a rate of ~50%. NF denotes glycopeptides that were ‘not found’ likely due to insufficient ionization.

**Figure S6: Glycans observed at each of 7 N-glycan sites on bacterial neuraminidase-treated unmodified rhGAA by LC-MS.** The schematics show the most abundant glycan structure found on each site in untreated and neuraminidase treated unmodified rhGAA and the tables show the relative abundance (%) of all other glycan structures found on each site in the neuraminidase treated sample. MS raw data were acquired using Xcalibur. Genedata Expressionist was used to process the raw data for glycopeptide identification. Glycan annotations are represented by symbols according to SNFG conventions in the schematic and Oxford notations in the tables. Site 7 in rhGAA is only occupied by glycan structures at a rate of ~50%.

**Figure S7: Glycans observed at each of 7 N-glycan sites on bacterial neuraminidase-treated oxidized rhGAA by LC-MS.** The schematic shows the most abundant glycan structure found on each site in untreated and neuraminidase treated oxidized rhGAA and the tables show the relative abundance (%) of all other glycan structures found on each site in the neuraminidase treated sample. MS raw data were acquired using Xcalibur. Genedata Expressionist was used to process the raw data for glycopeptide identification. Glycan annotations are represented by symbols according to SNFG conventions in the schematic and Oxford notations in the tables. “ox” denotes C7 oxidation of sialic acid. Site 7 in rhGAA is only occupied by glycan structures at a rate of ~50%.

**Figure S8: Glycans observed at each of 7 N-glycan sites on bacterial neuraminidase treated rhGAA-AOAA by LC-MS.** The schematics show the most abundant glycan structure found on each site in untreated and neuraminidase treated rhGAA-AOAA and the tables show the relative abundance (%) of all other glycan structures found on each site in the neuraminidase treated sample. MS raw data were acquired using Xcalibur. Genedata Expressionist was used to process the raw data for glycopeptide identification. Glycan annotations are represented b by symbols according to SNFG conventions in the schematic and Oxford notations in the tables. “ox” denotes C7 oxidation of sialic acid and blue line ( **|** ) denotes addition of AOAA to C7 oxidized sialic acid.

**B**

**A**

**Figure S9: Effect of glycogen size on GAA kinetics. (A)** Three commercial preparations of glycogen (from Sigma) were analyzed by gel filtration using a Superose 6 prep grade column (Cytiva and fractions subjected to phenol sulfuric acid assay to measure glycogen content colorimetrically. Weighted average molecular weights for the different glycogen samples were calculated using a standard curve obtained from running known samples of different molecular weights on the column. **(B)** Michaelis-Menten kinetics were performed using the three samples of glycogen incubated with precursor or mature unmodified rhGAA (lysates of Pompe patient fibroblasts after rhGAA uptake) for 60 min at 37°C. Data points were fitted to the Michaelis-Menten equation using Prism (GraphPad) and error bars represent mean ± SD.

**Table S2: Kinetics of glycogen hydrolysis.** Michaelis-Menten kinetics were performed using the three samples of glycogen incubated with precursor or processed rhGAA (lysates of Pompe patient fibroblasts after rhGAA uptake) for 60 min at 37°C (n=3 (6 total measurements on the Dionex ICS) for reactions containing low and high MW glycogen with precursor and processed rhGAA, n=7 (14 total measurements) for medium MW glycogen with precursor rhGAA, and n=5 (10 total measurements) for medium MW glycogen with processed rhGAA).

| **Glycogen relative MW and rhGAA form** | ***V_max_* ± SD**  **(μM/nM GAA/h)** | ***K_m_* ± SD**  **(μg)** | ***V_max_/K_m_*** | ***K_m_* ± SD**  ***(~mg/mL)*** |
| --- | --- | --- | --- | --- |
|  |  |  |  |  |
| Low MW glycogen - precursor | 638 ± 179 | 3314 ± 2191 | 0.193 | 21 ± 14 |
| Low MW glycogen - processed | 774 ± 113 | 1203 ± 624 | 0.643 | 8 ± 4 |
| Medium MW glycogen - precursor | 741 ± 370 | 9240 ± 7048 | 0.080 | 58 ± 44 |
| Medium MW glycogen - processed | 750 ± 168 | 2445 ±1367 | 0.307 | 15 ± 9 |
| High MW glycogen - precursor | 301 ± 145 | 21946 ± 13810 | 0.014 | 137 ± 86 |
| High MW glycogen - processed | 607 ± 345 | 9293 ± 8686 | 0.065 | 58 ± 54 |
